# Psychological proximity improves reasoning in academic aptitude tests

**DOI:** 10.1038/s41539-023-00158-x

**Published:** 2023-04-29

**Authors:** Britt Hadar, Maayan Katzir, Sephi Pumpian, Tzur Karelitz, Nira Liberman

**Affiliations:** 1 School of Psychological Sciences, Tel-Aviv University, Tel-Aviv, Israel; 2 Department of Psychology, Princeton University, Princeton, NJ USA; 3 Bar Ilan University, Ramat Gan, Israel; 4 The Hebrew University of Jerusalem, Jerusalem, Israel

**Keywords:** Human behaviour, Psychology

## Abstract

Performance on standardized academic aptitude tests (AAT) can determine important life outcomes. However, it is not clear whether and which aspects of the content of test questions affect performance. We examined the effect of psychological distance embedded in test questions. In Study 1 (*N* = 41,209), we classified the content of existing AAT questions as invoking proximal versus distal details. We found better performance with proximal compared to distal questions, especially for low-achieving examinees. Studies 2 and 3 manipulated the distance of questions adapted from AATs and examined three moderators: overall AAT score, working-memory capacity, and presence of irrelevant information. In Study 2 (*N* = 129), proximity (versus distance) improved the performance of low-achieving participants. In Study 3 (*N* = 1744), a field study, among low-achieving examinees, proximity improved performance on questions that included irrelevant information. Together, these results suggest that the psychological distance that is invoked by the content of test questions has important consequences for performance in real-life high-stakes tests.

Consider the following problem: Danielle prefers bananas over apples, and apples over strawberries. Considering her parents want to send her favorite fruit in the lunchbox, should they buy bananas or strawberries? This type of problem is often presented in academic aptitude tests (AAT), but can also emerge in real life, when, for example, we need to reason through a social situation (e.g., reach an integrative agreement between multiple parties with conflicting interests), or come up with a novel, creative way to formalize a multifaceted problem.

The present paper examines how psychological distance affects reasoning with this type of questions. The psychological distance to an object is the extent to which a person feels that it is removed from “the reality of me, here and now” in either time (future or past), space, social perspective, or the realm of hypothetical worlds. Construal level theory (CLT) extensively studied the effects of psychological distance on the way information is represented and processed^1–4^. Specifically, CLT proposes that objects that are distal in time, space, or social perspective or are uncertain (i.e., distant on the dimension of hypotheticality), are mentally represented (i.e., construed) on a higher level of abstraction. Higher level, abstract mental representations extract the gist of informational input by retaining those features of input stimuli that the perceiver deems essential, whereas low-level construals include also incidental, contextual features of the input.

In the present set of studies we examined whether it would help examinees to arrive at the correct solution in reasoning questions if the problems pertain to psychologically distal objects and events (in the example above, make it about more exotic fruits such as persimmon, pitaya, and guava), or rather if the problems pertain to objects and events in the solver’s environment (in the example above, make the problem about familiar fruits, retaining its logical structure)? It is important to note that although there might be a potential utility of prior knowledge in problem-solving, the prior knowledge activated by proximity or distance is superfluous to solving the problem correctly. For example, the arithmetic problem “2 + 2” is not a-priori facilitated by knowledge of whether the items being counted are apples, bananas, persimmons, or pitaya; the physical characteristics of these fruits are extraneous to the arithmetic calculation. To perform the calculation, one does not rely on any known properties of the fruit. We designed our studies to investigate whether this extraneous aspect of the problem, specifically, whether the objects in the problem are psychologically proximal or distal, would affect performance.

The literature seems to offer opposite answers. Some approaches suggest that distancing would make it easier for the problem solver to filter out irrelevant details (e.g., the distracting thought “won’t strawberries get smashed in the lunchbox?” is less likely to occur with respect to a pitaya if one has, as most of us, no clue how it looks), and thus represent the logical structure of the problem more clearly. In contrast, other approaches suggest that proximity would make the problem details more concrete and vivid and thus make it easier to represent the problem parameters and to ultimately solve it (e.g., being able to represent strawberries and apples in shape and color helps one represent the relations between them).

The question of whether distance or rather proximity is more conducive to solving reasoning problems is obviously interesting for practical reasons, because knowing the answer would help people solve such problems, and design interventions to help others. But the question is also interesting for theoretical reasons, in view of the aforementioned disagreement. In what follows, we first review in more detail the opposing views on how distance might affect solving reasoning problems, and then report three studies that attempted to answer it empirically.

## Psychological distance improves reasoning about complex problems by facilitating abstraction

Many theoretical traditions seem to suggest that distancing a problem from oneself, here-and-now improves reasoning. For example, DeLoache^5^ found that physically distancing children from maps or models (e.g., by placing the map/model behind a window) helped them to solve problems that required the understanding of the relationship between the symbol and its referent (i.e., the model and the room that it represents). In contrast, increasing proximity to the model (e.g., by allowing children to play with the model) impaired understanding of the mapping between the model and its referent and interfered with problem solving^5–8^.

Distance has also been found to increase creative problem solving in both children^9^ and adults^10–13^. For example, in one of their studies, Polman and Emich^12^ showed that people solved a problem better when taking a socially distant perspective (imagined themselves solving the problem on behalf of someone else) compared to a proximal perspective (imagined themselves being immersed in the problem). As another example, studies on negotiation have shown that taking a distant perspective (e.g., assuming physical or temporal distance from the focal event) improved the chances of the parties to reach integrative, mutually beneficial agreements about complex, multi-dimensional problems^14–16^.

Within CLT^1–4,17^, psychological distance has been theorized and found to foster more abstract representations of objects. Hence, findings that show that more abstract (versus concrete) representations of problems facilitate their solution e.g.,^18–20^ are consistent with the possibility that psychological distancing would have a similar effect.

Indeed, abstraction has been proposed to mediate and explain the beneficial effects of psychological distance on problem solving e.g.,^6,7^. More specifically, abstraction engenders two distinct yet related processes (see Gilead et al., 2020 for a review of the functions of abstraction): One process is filtering out of irrelevant information, such that distracting or irrelevant details are omitted, making it easier to concentrate on the critical parameters of the problem^21^. The second process is integration of information, such that objects are grouped together to form a broader category^22,23^. Both processes might assist mental operations that are critical for solving complex problems. Specifically, both filtering and grouping may reduce load on working memory^24,25^, a resource that is critical for solving complex problems, for review, see^26^.

The potential interaction between psychological distance, working memory capacity, and the presence of irrelevant information could manifest in a number of ways. For instance, when individuals are not immersed in the vivid details of a problem—i.e., they are psychologically distant from it—they may be less likely to be distracted by irrelevant information. Consequently, they may be able to focus more effectively on the problem’s critical aspects, thereby reducing the cognitive load on their working memory. Furthermore, psychological distance can facilitate categorization by enabling individuals to group related information into broader categories^23^. This, in turn, can help simplify mental organization and processing, further reducing the demands on working memory ^27^.

## Psychological proximity improves reasoning about complex problems by increasing vividness and attention to detail

Of course, solving complex problems does not rely solely on abstraction, but may also be assisted by a clear, vivid representation of the problem with all its relevant detail. It has been found that stimuli that are more proximal to me, here-and-now are processed with more attention, are represented in more detail, and are remembered better e.g.,^28^, and hence, to the extent that problem solving relies on these processes, psychological proximity should assist it.

Several findings support this notion. For example, when objects were presented within physical reach, people segregated a figure from its background more accurately^29^, were less prone to visual illusions^30^, exhibited improved letter recognition^31^, and faster discrimination between shapes^32^. Socially proximal information (about oneself or close others as opposed to about unfamiliar people) has also been shown to have similar advantages for cognitive processing: people remember information related to them and to objects they own better than information related to others and their objects^33^, an effect coined the self-reference effect in memory^34^, for a review and meta-analysis see^35–37^. Psychological proximity (compared to distance) also improved peoples’ performance on problems that required them to find missing parts in detailed pictures (e.g., a picture of a man with a watch, but the watchband is missing from it)^38^, Study 6.

Why are proximal objects processed better? Neuroscientific research suggests one reason according to which information presented in the space near the body (“peripersonal space”) receives preferential processing^39^, for a review, see^40^. It has also been suggested that proximal objects tend to attract more attention due to their relevance, that is, because when proximal, they can, for example, be consumed, manipulated, pose danger, or yield reward more than when distal^41^. Within the framework of CLT, proximity engenders more concrete, vivid and detailed representations. Thus, according to CLT, proximity should facilitate solving of problems that rely on these processes.

Consistent with the facilitating effects of proximity are findings that concrete, embodied processing of problems (compared to abstract processing) assist in solving them, for a review see^42^. A meta-analysis of 55 studies on students from kindergarten to college showed that the use of concrete objects (compared to abstract symbols) at math instruction facilitated problem solving, retention, and transfer of learned materials to new domains^43^.

Moreover, psychological proximity may enhance problem solving by influencing working memory. When an individual is psychologically close to a problem (e.g., they are well immersed in the vivid details), they may be recruiting more memory traces from long-term memory to support information currently held in working memory^44^. Furthermore, the familiarity associated with psychologically proximal objects or events may decrease anxiety, which can free up working memory space^45^. Collectively, these processes may result in better problem-solving.

## The present research

In light of the aforementioned disagreement, our aim was to examine how psychological distance that is embedded in the problem parameters would affect performance on reasoning problems. We used verbal reasoning questions, because they require both an accurate representation of the specific parameters of the problem, and an accurate mapping of these parameters on an abstract logical structure. To better understand the effect of distance, we tested three moderators of its effect: overall AAT performance (Studies 1–3), working-memory capacity (Study 2), and presence of irrelevant details in the test question (Studies 2–3). All three moderators were expected to be related to performance (performance on the target items was expected to be higher among high-scoring individuals, among individuals with higher working-memory capacity and with questions that include only relevant details). In addition, as noted above, different theoretical approaches predict interactions of these parameters with the effect of distance. In particular, if distance or higher working memory capacity facilitate performance by making it easier to filter-out irrelevant details, then their effect might be more pronounced in problems in which such details are present.

**Fig. 1:**
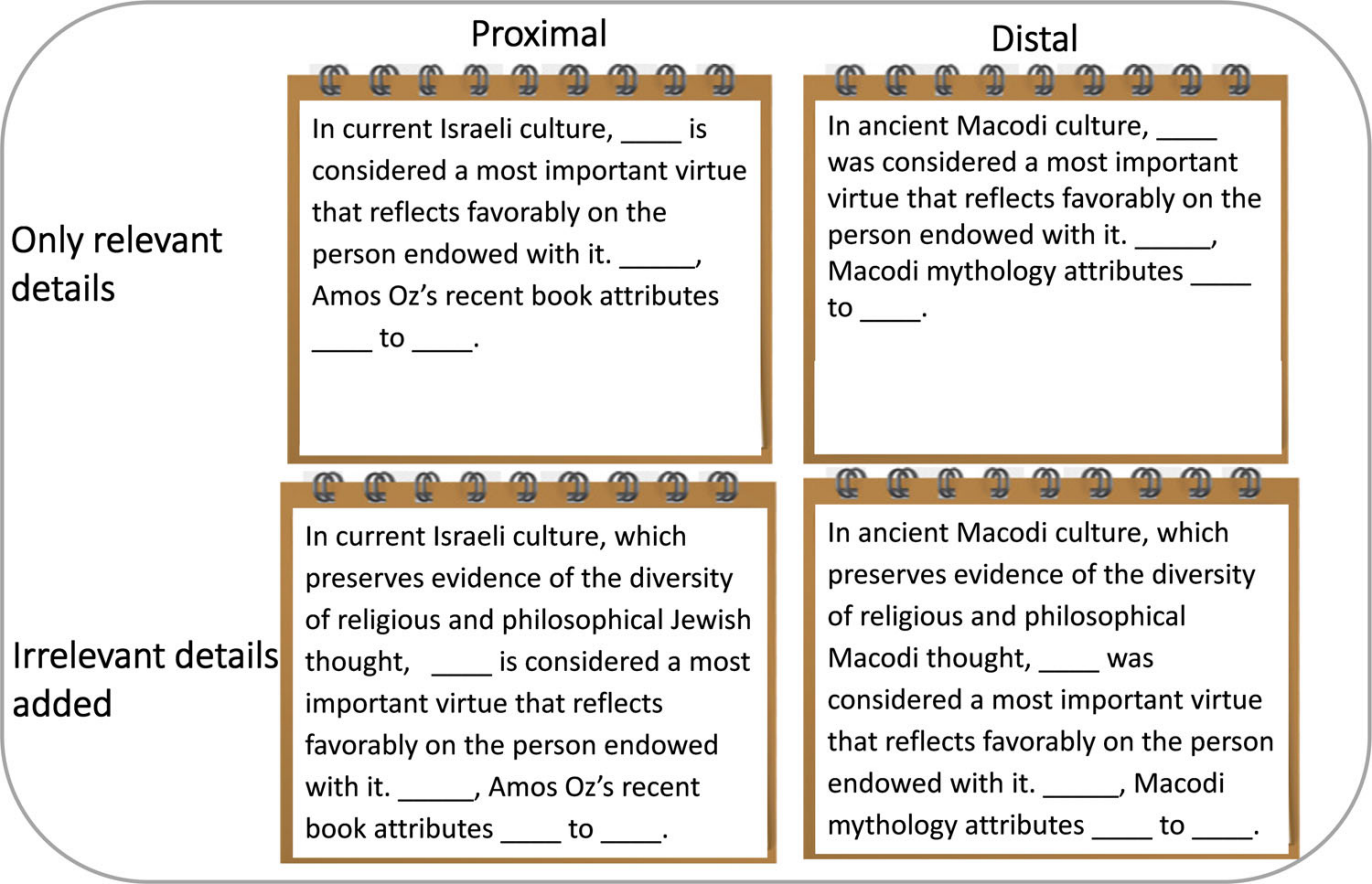
Sample question in all four versions. A test question in all four versions, comprising a distance (proximal vs. distal) by relevance of details (relevant-only vs. irrelevant-added) design (Studies 2 and 3). Each participant was presented with one of the four versions of each question. Distance was manipulated between participants (i.e., a participants answered all questions in either the proximal or the distal version) whereas relevance was manipulated within participants (i.e., each participant received the relevant-only version of six questions, and the irrelevant-added version of other six questions). Each question was followed by four alternative responses, of which participants chose the best answer: (1) composure / Yet / the ability to face danger without batting an eyelid / the villain Carvilius (Molko), of all people. (2) kindness / Yet / the willingness to help the needy at any time / the hero Carvilius (Molko), of all people. (3) modesty / Indeed / unparalleled arrogance / the revered hero Carvilius (Molko). (4) physical strength / Indeed / great physical power / the notorious villain Carvilius (Molko).

In three studies, we examined performance on questions that included details that were either psychologically proximal or psychologically distal in time, space, and on the social dimension. Specifically, in the proximal condition, the questions were about objects and places in the participants’ culture and environment, about members of their ingroup and events in the recent past. In contrast, in the distal condition, questions were about objects and places from distant cultures, members of unfamiliar groups, and events in the distant past (see Fig. 1).

## Transparency and openness

The method section describes the sample, all data exclusions (if any), all manipulations, and all measures in the study. All data and analyses code are available at Open Science Repository (osf.io/2nvtq). Data were analyzed using R, version 4.1.2 and the package ggplot, version 3.3.5^46^. This study’s design and its analyses were not preregistered. The methods were performed in accordance with relevant guidelines and regulations and approved by the Institutional Review Board of Tel Aviv University.

## Analyses

In all studies we ran a generalized linear mixed model (GLMM) using the glmer function (family = binomial) in the lme4 package in R version 1.1-30^47^. Accuracy was coded as a binary variable (1 = correct, 0 = incorrect). AAT scores were mean-centered^48^, and categorical variables were effect-coded (1 = distal, −1 = proximal; 1 = irrelevant, −1 = relevant). Type-III significance levels were calculated with the ‘car’ package version 3.1-0 ^49^.

## Results

Study 1

Study 1 examined whether performance would be better with psychologically proximal (vs. distal) questions. We also included examinees’ final AAT scores as factor in the analysis. This enabled us to evaluate whether the effects of psychological distance vary according to ability level. We collected data from all publicly available AATs administered between the years 2014–2017 (*N* = 41,209). We analyzed accuracy on verbal reasoning questions that were distal versus proximal as coded by judges. We obtained information on performance on these questions from the organization that administered the test.

**Fig. 2:**
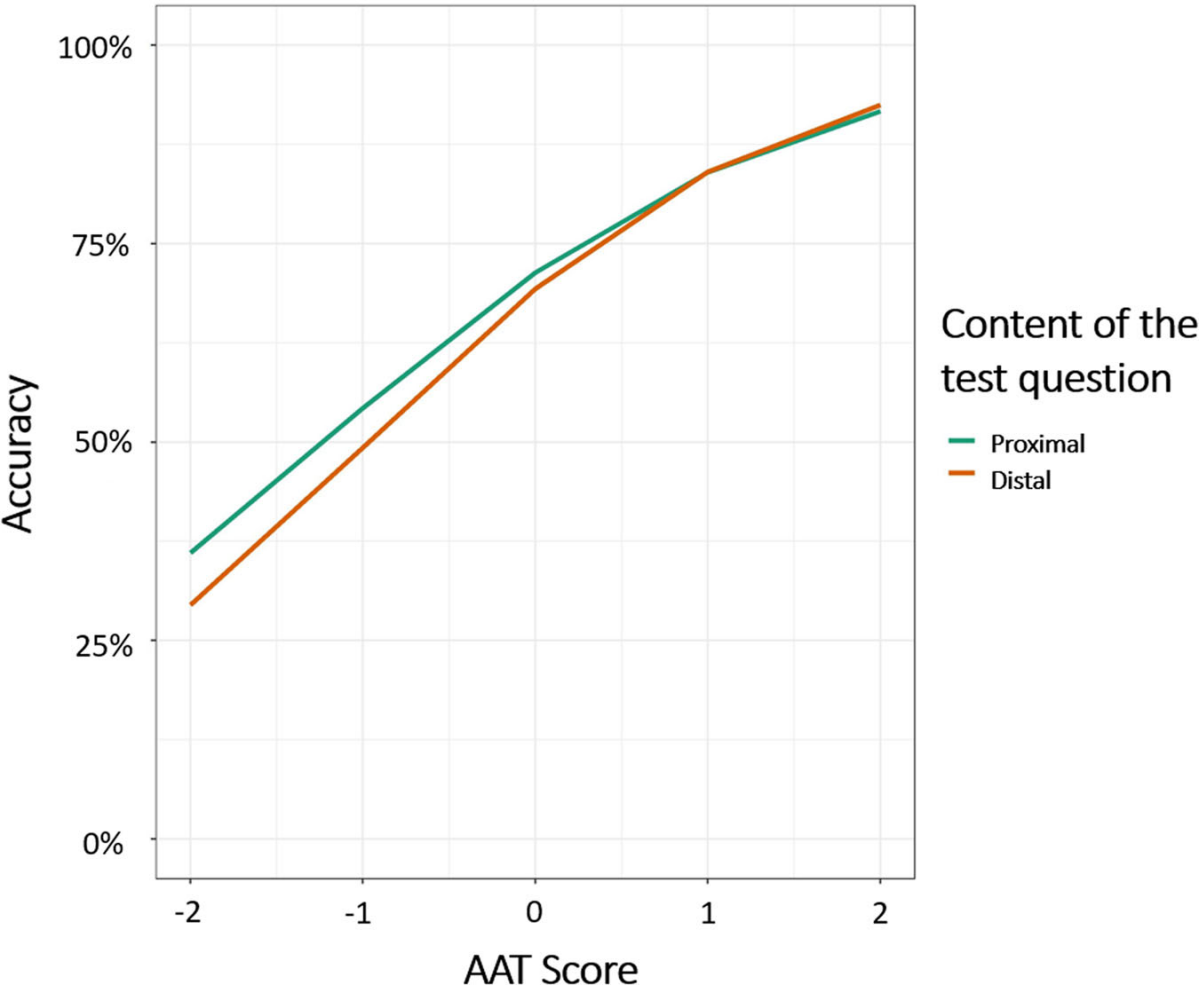
Results of study 1. Accuracy as a function of AAT (in standard deviation scores) and distance of the test question.

We examined the effect of distance and AAT score on accuracy. As expected, accuracy was higher for participants with higher AAT scores, *B* = 0.793, *SE* = 0.07, *z* = 108.49, *p* < 0.0001. Critically, accuracy was higher with proximal than with distal questions, *B* = −0.049, *SE* = 0.007, *z* = −6.80, *p* < 0.0001, (*M*_*-proximal*_ = 0.71, *SE*_*-proximal*_ = 0.01, *M*_*-distal*_ = 0.68, *SE*_*-distal*_ = 0.01). An interaction between AAT and distance, *B* = 0.05, *SE* = 0.007, *z* = 7.64, *p* < 0.0001, indicated that the advantage of proximal questions over distal questions was more pronounced for low-achieving examinees (whose AAT scores were lower than the average), see Fig. 2. We present the regression coefficients in Table 1.

**Table 1:** Regression analyses for Study 1.

Predictors	Estimate	Standard Error	Odds Ratio	CI	*p*
(Intercept)	0.865	0.252	2.38	1.46–3.89	**0.001**
AAT	0.793	0.007	2.21	2.18–2.24	**<0.001**
Distance	-0.049	0.007	0.95	0.94–0.97	**<0.001**
Distance * AAT	0.050	0.007	1.05	1.04–1.07	**<0.001**

Notes. Academic aptitude test score (AAT), distance (−1 = proximal, 1 = distal).

Values in bold indicate statistically significant effects.

Study 2

In study 1 we compared to each other questions that could have had different logical structure. Study 2 (*N* = 129) aimed to examine the effects of distance on verbal reasoning while keeping the logical structure of the question constant. We used materials from a standardized AAT, and adjusted the content of the questions to refer to distant or proximal objects and situations (between participants), and to include only relevant or also irrelevant details (within participants). We also explored the possible moderating effects of AAT and of working-memory capacity on the effects of distance, relevance, and their interactions. We hypothesized that (a) AAT scores would positively correlate with performance, (b) working-memory capacity would positively correlate with performance, (c) adding irrelevant details would impair performance (compared to not adding them), and (d) replicating Study 1, proximity would enhance performance, especially for participants with low AAT scores.

As both AAT scores and working-memory capacity reflect cognitive ability, they tend to be positively associated e.g.,^50^. In the current sample, however, only a modest, insignificant correlation emerged, *r*(110) = 0.16, *p* = 0.087, possibly due to the restricted range of AAT scores which could have reflected the high admission threshold of students in the psychology program of the relevant university (in our sample: *M* = 652, *SD* = 62; the grand mean in the relevant population is *M* = 579, *SD* = 107). We therefore included both AAT score and working-memory capacity (*K*_*max*_) in the same model. We present the regression coefficients in Table 2.

**Table 2:** Regression analyses for Study 2.

Predictors	Estimate	Standard Error	Odds Ratio	CI	*p*
(Intercept)	0.318	0.221	1.37	0.89–2.12	0.150
AAT	0.349	0.135	1.42	1.09–1.85	**0.009**
K (working-memory capacity)	0.297	0.147	1.35	1.01–1.80	**0.044**
Distance	−0.289	0.199	0.75	0.51–1.11	0.147
Relevance	−0.113	0.168	0.89	0.64–1.24	0.499
AAT * K	0.315	0.150	1.37	1.02–1.84	**0.035**
AAT * Distance	0.536	0.235	1.71	1.08–2.71	**0.022**
K * Distance	−0.379	0.201	0.68	0.46–1.02	0.060
AAT * Relevance	0.022	0.167	1.02	0.74–1.42	0.897
K * Relevance	−0.240	0.181	0.79	0.55–1.12	0.183
Distance * Relevance	0.008	0.245	1.01	0.62–1.63	0.975
AAT * K * Distance	−0.130	0.239	0.88	0.55–1.40	0.585
AAT * K * Relevance	0.297	0.184	0.74	0.52–1.07	0.107
AAT * Distance * Relevance	−0.352	0.281	0.70	0.41–1.22	0.210
K * Distance * Relevance	0.482	0.250	1.62	0.99–2.64	0.054
AAT * K * Distance * Relevance	−0.182	0.290	0.83	0.47–1.47	0.529

Notes. Academic aptitude test score (AAT), working-memory capacity estimate (K), distance (−1 = proximal, 1 = distal), relevance (−1 = relevant, 1 = irrelevant).

Values in bold indicate statistically significant effects.

**Fig. 3:**
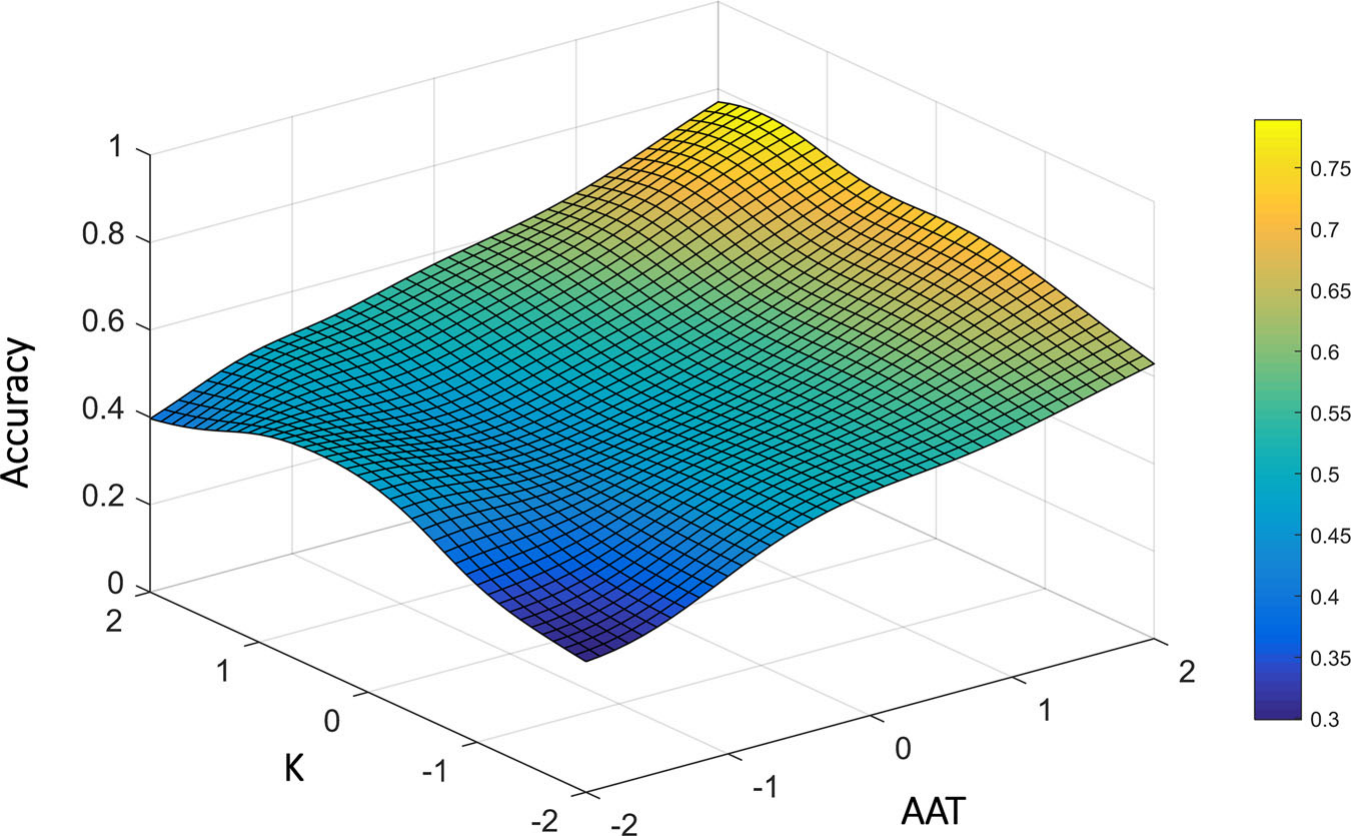
Results of study 2. Accuracy as a function of AAT score and working-memory capacity (*K*_*max*_).

We assessed the effect of distance, relevance, AAT score, *K*_*max*_ and their interactions on accuracy. As expected, accuracy was higher for participants with higher AAT scores, *B* = 0.349, *SE* = 0.135, *z* = 2.59, *p* = 0.009, as well as for participants with higher working-memory capacity, *K*_*max*_, *B* = 0.297, *SE* = 0.147, *z* = 2.01, *p* = 0.044. An interaction between these scores, *B* = 0.315, *SE* = 0.150, *z* = 2.103, *p* = 0.035, indicated that their effects intensified each other, namely, with higher AAT scores, working-memory capacity had a larger effect on accuracy, and that with higher *K*_*max*_, AAT scores had a larger effect on accuracy (Fig. 3).

Distance did not significantly affect accuracy, *B* = −0.289, *SE* = 0.199, *z* = −1.45, *p* = 0.147, (*M*_*-proximal*_ = 0.57, *SD*_*-proximal*_ = 0.19, *M*_*-distal*_ = 0.49, *SD*_*-distal*_ = 0.21), nor did relevance, *B* = −0.113, *SE* = 0.168, *z* = −0.67, *p* = 0.499, (*M*_*-relevant*_ = 0.55, *SD*_*-relevant*_ = 0.24, *M*_*-irrelevant*_ = 0.51, *SD*_*-irrelevant*_ = 0.24). The only effect that involved relevance was a marginally significant three-way interaction between relevance, distance and *K*_*max*_, *B* = 0.482, *SE* = 0.250, *z* = 1.93, *p* = 0.054. Inspection of the interaction revealed that adding irrelevant information impaired the performance only among participants with low *K*_*max*_ in the distal condition, *B* = 0.284, *SE* = 0.135, *z* = 2.10, *p* = 0.035.

**Fig. 4:**
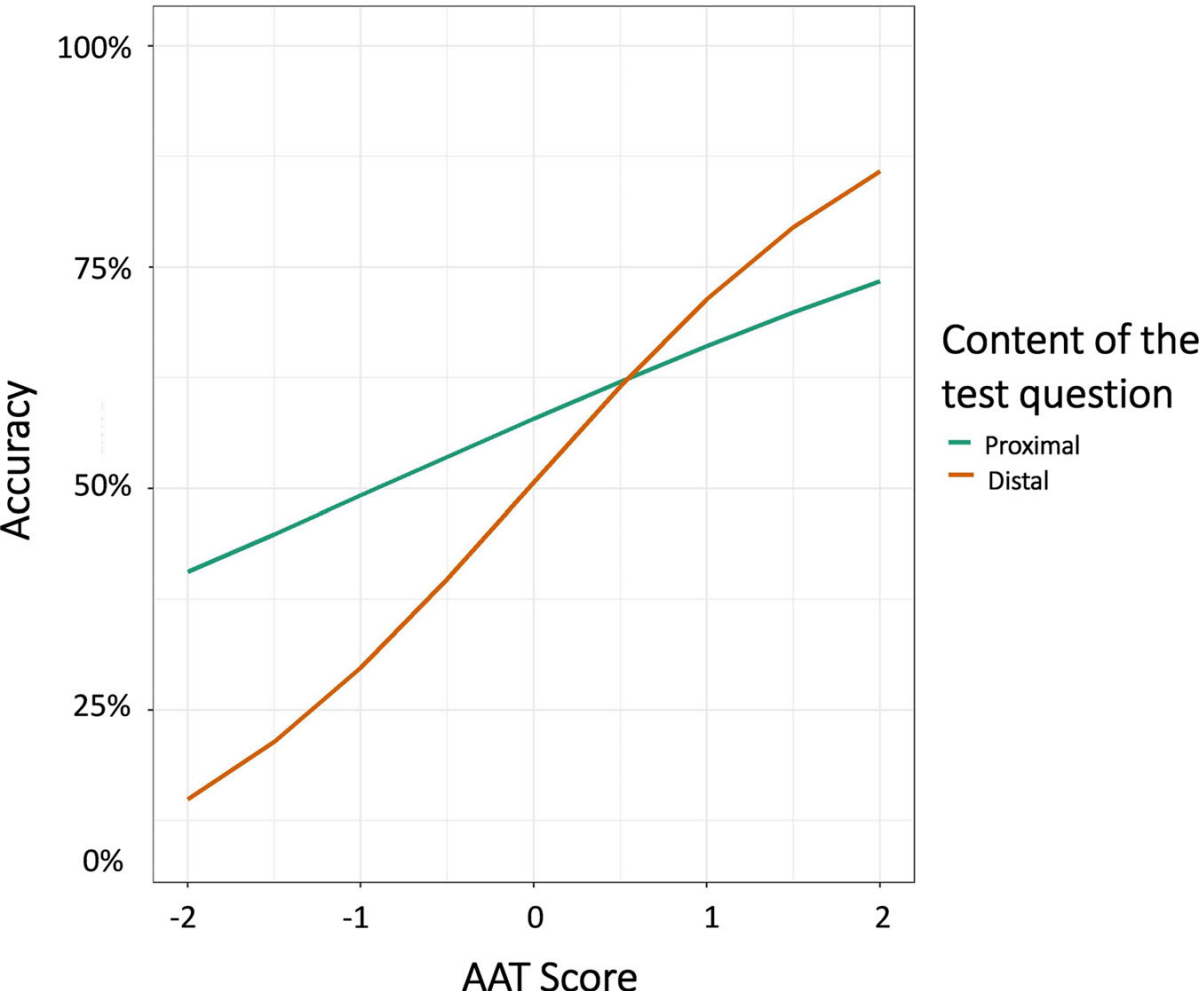
Results of study 2. Accuracy as a function of AAT score (in standard deviations scores), and distance.

Importantly, replicating the findings of Study 1, an interaction between distance and AAT score, *B* = 0.536, *SE* = 0.235, *z* = 2.28, *p* = 0.022, indicated an advantage of proximal questions over distal questions among low-achieving participants but not among high-achieving participants. The effect of distance for participants with below-average AAT score was quite sizeable: accuracy with proximal questions (*M* = 0.50) was 28% higher than with distal questions (*M* = 0.39), although proximal and distal questions only differed in content and had the same logical structure (Fig. 4).

The interaction of AAT with relevance (*p* = 0.89), and the three-way interaction between AAT, distance and relevance (*p* = 0.21), were not significant.

Study 3

Study 3 (*N* = 1744), aimed to replicate Study 2 in an ecological settings, with a large, representative sample, and a broader range of AAT scores. We used the context of a consequential high-stakes exam that would determine individuals’ admission chances to higher-education institutions. By looking at performance in this high-stakes, real life settings we aimed to increase the generalizability and reliability of the results by minimizing the extent to which the findings are attributable to low motivation or to a subset of participants who have been admitted to selective higher-education institutions. To that end, we collaborated with an organization that administers nation-wide academic aptitude tests, and incorporated the experimental questions into the exam administered as an entrance requirement for higher-education institutions. As in Study 2, we manipulated distance (proximal vs. distal) between participants and relevance (relevant-only vs. irrelevant-added) within participants. Based on the results of Studies 1–2, we predicted that proximity would enhance performance, especially for participants with low AAT scores.

We examined the effect of distance, relevance, AAT score, and their interactions on accuracy (see Table 3). The analysis revealed that as expected, accuracy was higher for participants with higher AAT scores, *B* = 0.823, *SE* = 0.024, *z* = 33.72, *p* < 0.001. Distance did not affect accuracy, *B* = −0.037, *SE* = 0.023, *z* = −1.601, *p* = 0.109 (*M*_*-proximal*_ = 0.66, *SD*_*-proximal*_ = 0.47, *M*_*-distal*_ = 0.64, *SD*_*-distal*_ = 0.48). Adding irrelevant information had a marginal negative effect on accuracy, *B* = 0.076, *SE* = 0.039, *z* = −1.92, *p* = 0.055, (*M*_*-relevant*_ = 0.67, *SD*_*-relevant*_ = 0.47, *M*_*-irrelevant*_ = 0.64, *SD*_*-irrelevant*_ = 0.48).

**Table 3:** Regression analyses for Study 3.

Predictors	Estimate	Standard Error	Odds Ratio	CI	*p*
(Intercept)	0.838	0.161	2.55	1.88–3.45	**<0.001**
Wave	−0.011	0.151	0.99	0.74–1.33	0.940
AAT	0.823	0.024	2.33	2.14–2.54	**<0.001**
Distance	−0.037	0.023	0.96	0.85–1.07	0.109
Relevance	−0.076	0.039	0.88	0.75–1.05	0.055
AAT * Distance	0.005	0.023	0.92	0.82–1.04	0.817
AAT* Relevance	0.019	0.020	0.94	0.85–1.05	0.333
Distance * Relevance	−0.014	0.019	0.95	0.81–1.10	0.455
AAT * Distance * Relevance	0.047	0.019	1.21	1.04–1.41	**0.015**

Notes. Wave indicates the two occasions in which the test was administered nationally and data was collected, academic aptitude test score (AAT), distance (−1 = proximal, 1 = distal), relevance (−1 = relevant, 1 = irrelevant).

Values in bold indicate statistically significant effects.

**Fig. 5:**
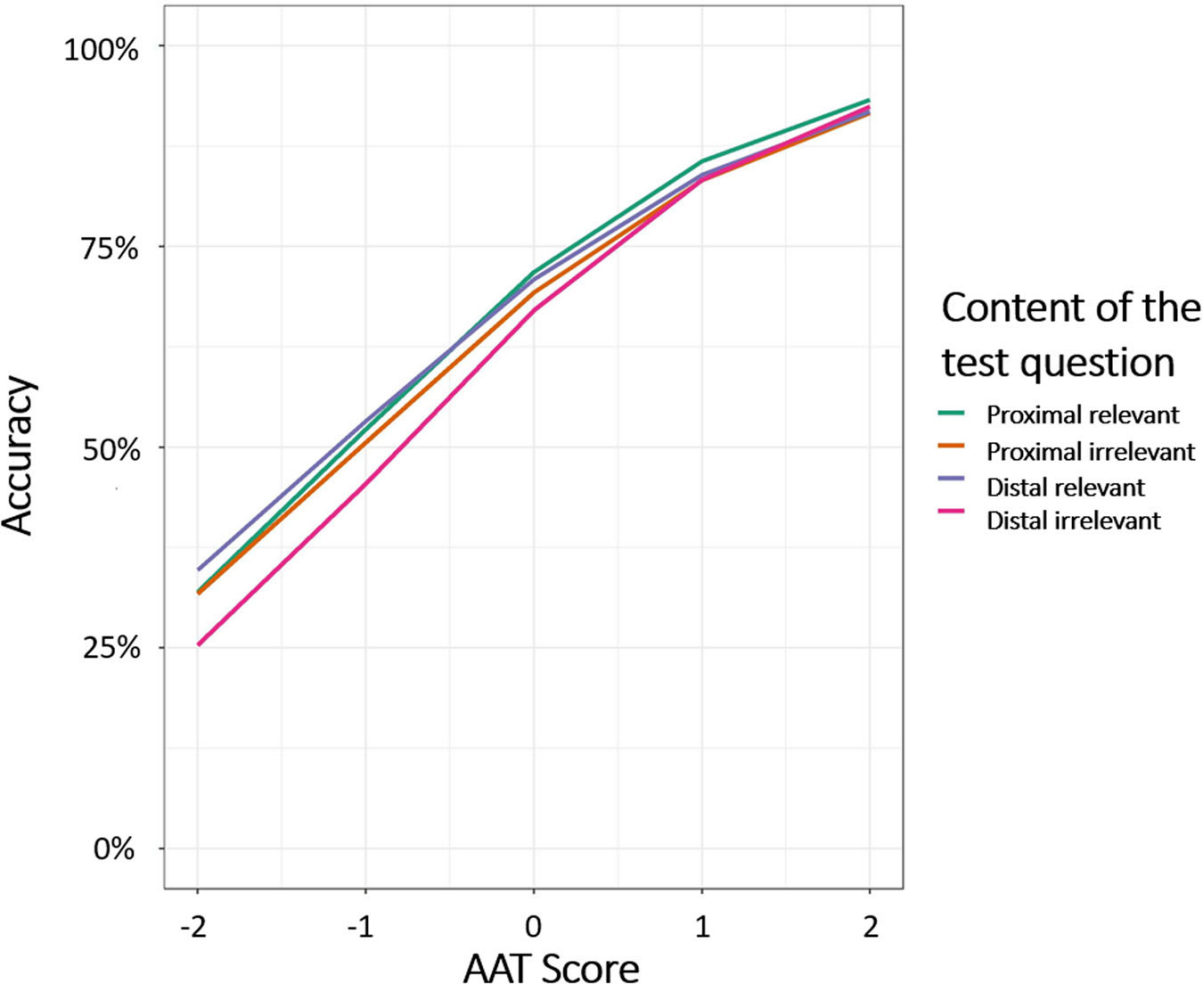
Results of study 3. Accuracy as a function of AAT score (in standard deviation scores), relevance, and distance.

Although the interaction between distance and AAT score was not significant, *B* = 0.005, *SE* = 0.023 *z* = 0.232, *p* = 0.817, a three-way interaction between distance, AAT, and relevance emerged, *B* = 0.047, *SE* = 0.019, *z* = 2.43, *p* = 0.015, (Fig. 5). A follow-up analysis revealed that whereas the performance of high-achieving examinees was not affected by either relevance (*p* = 0.503), distance (*p* = 0.890), or their interaction (*p* = 0.880), the performance of low-achieving examinees was impaired by adding irrelevant information *B* = −0.146, *SE* = 0.050, *z* = −2.91, *p* = 0.003. Also in this group, although distance did not affect performance as a main effect, (*p* = 0.390), it interacted with relevance, *B* = −0.087, *SE* = 0.040, *z* = −2.16, *p* = 0.031, such that accuracy was lower in distal-irrelevant questions, *B* = −0.137, *SE* = 0.063, *z* = −2.17, *p* = 0.030. For participants with below-average AAT score, performance on proximal questions (*M* = 0.52) was higher compared to distal questions (*M* = 0.48) by 7%, but only when the questions were made more difficult by including irrelevant details.

## Discussion

Across three studies - in a corpus of large-scale real-life data attained from an academic aptitude test (AAT; Study 1), in the lab (Study 2), and in the field (Study 3), we examined how performance on verbal reasoning questions is affected by the examinee’s psychological distance from the content of those questions. In all studies, individuals completed verbal reasoning questions from standardized AATs. The questions were about objects that were either psychologically distal or psychologically proximal to the examinees in time, space, and social distance. In Study 1, questions included only information necessary for solving the problem, whereas in Studies 2 and 3 some of the questions also included additional, irrelevant details.

In Study 1 we found that people scored higher on questions that presented proximal content compared to questions that presented distal content. Notably, the facilitative effect of proximity was more pronounced among low-achieving examinees than among high-achieving examinees. In Study 2, we found the same pattern: proximity (compared to distance) enhanced performance for participants who attained low scores on the AAT. In Study 3, we predicted that proximity would improve the performance of low-achieving examinees (as in Studies 1–2), but we found this to be true only in questions that were made more difficult by including irrelevant details. Our results, therefore, suggest that proximity is especially advantageous (or, conversely, distance is most detrimental) for low-achieving individuals and when difficulty is high. In Studies 1 and 2 proximity facilitated correct responding among examinees who attained below-average AAT scores, and in Study 3 proximity facilitated correct responding on irrelevant-added problems among examinees who attained below-average AAT scores. Indeed, the average accuracy rate for low-achieving examinees (those with 1 SD below the mean) in Study 1 (*M* = 0.43), and in Study 2 (*M* = 40), and for low-achieving examinees in the irrelevant-added problems in Study 3 (*M* = 0.42) was similar. In this way, the differences between the results obtained in the three studies may in fact point to a consistent pattern - whereby proximity is facilitative (or distance is detrimental) when difficulty is high. Importantly, these effects emerged not only in the lab, but also when participants took a consequential, high-stakes exam that could determine their admission chances to higher-education institutions, and possibly affect their future career paths.

These findings are in line with previous research which documented that proximity enhanced cognitive performance, for a review, see^28^, and extend this research in several ways. In particular, the present research focuses on conceptual distance, which is ubiquitous in any object or event as they would be inevitably described by the examiner (or imagined by the examinee) to be at some distance – to occur in a certain proximal or distal point in time and space, to be familiar to the examinee or their social group, or, conversely to denote a socially distal group.

Psychological distance to test items is idiosyncratic: It may depend on the level of familiarity, knowledge and exposure to various objects, people and situations that are mentioned in the item. However, some differences in a person’s distance to test items may also depend on social group. Culture-specific entities such as public figures, symbols, historical events or heritage sites would be typically regarded as close and familiar for certain people while distant and alien for others. Our results suggest that the extent to which an examinee feels proximal to the content of a question can affect performance. For example, the extent to which an examinee feels close to the British philosopher David Hume can determine how well they do on a question that invokes this historical intellectual figure. We thus suggest that test developers take into account the demographic and socioeconomic characteristics of examinees, and try to construct questions that do not create or amplify differences in level of achievement that are based on these differences. This could be achieved by pre-testing the materials and tailoring the content to avoid making it psychologically closer to some examinees than to others. Our findings thus identify a new source of potential cultural bias in AAT questions. The finding that low-scoring examinees are more affected by distance is also informative in this regard, as it suggests that this cultural bias would impact this segment in particular.

Why does proximity improve reasoning? Some of the features that characterize proximal stimuli have been found to facilitate cognitive performance. For example, people are usually more familiar with proximal objects than with distal objects, and studies have found that familiar objects are remembered better than unfamiliar ones^51,52^, possibly because familiar objects rely on long-term memory processes that shield representations from interference^44^. Moreover, people tend to physically hold, manipulate, and interact with proximal objects more often than with distal objects and thus would tend to have a more embodied representation of them^53–55^. According to embodied cognition theory, cognition has evolved to facilitate action, and as such, cognitive processes that support action are more accurate and efficient. It is possible, then, that reasoning is better with proximal objects because they are more easily embodied. Also, a meta-analysis of 19 neuroimaging studies^56^ showed that the verbal system is involved to a greater extent with the processing of abstract rather than concrete concepts, while the perceptual system is involved to a greater extent with processing of concrete rather than abstract concepts. To the extent that mental imagery or other perceptual simulation facilitate reasoning about a problem, concrete (rather than abstract) representation might be more conducive for performance. Future research, however, is needed to determine what are the conditions in which vivid mental imagery would assist problem solving.

Social psychology suggests another potential mediator for the effect of psychological distance, namely, the possibility that distal content might communicate to some examinees that they do not belong to the relevant social environment. For example, it is possible that questions about David Hume, about distal planets and even about a brioche might suggest that “anybody who is not familiar with these things does not belong here”. Studies on stereotype threat have found that performance is undermined when (and to the extent that) difficulty suggests to students that they do not belong to the current social environment^57^. These processes have been shown to reduce working memory capacity and negatively affect cognitive performance^58,59^.

From a practical perspective, our findings can inform users and developers of cognitive tests. Tasks akin to the one used in our studies are integral to many cognitive assessment tests, such as intelligence tests (e.g., Wechsler intelligence scales)^60^, aptitude tests (e.g., scholastic assessment tests; SAT), cognitive functioning tests (e.g., MoCA)^61^; and even skills assessment tests in job interviews. Accordingly, it is imperative to understand how performance is affected by seemingly neutral aspects of test questions, such as whether they pertain to distal or proximal objects.

## Methods

Study 1

### Participants

Participants were 41,209 examinees taking the AAT. Overall, we collected data from 10 exams, see Table 4. Participants signed informed consent as part of the enrollment process to the test.

**Table 4:** Participants in Study 1.

Exam	*N*	Number of Items	Age	%Women
2014a	4602	3	21.75	62.7
2014b	5810	4	20.35	53.3
2014c	5579	2	21.94	59.9
2015a	2760	2	22.47	49.8
2015b	5815	2	19.73	52.6
2015c	3981	8	21.27	57.3
2016a	2947	3	22.51	57.7
2016b	3546	4	19.64	50.2
2017a	2522	5	22.14	53.4
2017b	3647	5	21.56	54.3

Notes. Each exam was administered in several waves each year - the letters indicate the waves’ order in each year. N represents the number of examinees in the analysis. Number of items indicates how many test questions from each exam were analyzed. Information on age and gender was obtained from a demographic form that examinees completed on a voluntary basis at the end of each exam.

### Materials and procedure

Examinees underwent the AAT, a test administered nationally, in which each examinee received a test form, comprised of three sections in the domains of quantitative reasoning, verbal reasoning, and English. Examinees also complete a writing assignment. Each of the three sections has 20–23 multiple-choice questions, which the examinee has 20 minutes to complete. We selected questions from the verbal reasoning section which were coded by two judges (*r* = 0.88) as either proximal or distal, with a total of 38 unique questions (*M_accuracy* = 0.66, *SD_accuracy* = 0.19).

The following is an example of a distal question, including the multiple-choice options: “In the year 1952, the following appeared in a literary magazine: “In a 1940 interview, the famous Romanian author M. Petreu announced that he would no longer be writing children’s books, but would spend his time writing poetry. While Petreu has indeed stopped writing children’s books, he has not yet published a book of poetry”. Assuming that what was written in the magazine is true, which of the following is impossible? (1) All of M. Petreu’s books are children’s books; (2) M. Petreu published his first book of poetry in 1953; (3) All of M. Petreu’s books are books of poetry; (4) M. Petreu published nothing after 1940.”

An example of a proximal question is: “In recent years a new, mechanized method for separating the seeds of the corn from its pulp was developed. This led to a reduction in the price of corn seeds and their sales rose significantly. As a result, the number of corns grown in the US increased greatly. However, a problem arose: the amount of waste – the corn pulp – that accumulated in the factories separating the seeds grew tremendously. A new study found that adding corn pulp to the fodder for sheep and cows greatly improved their health and the quality and quantity of the milk they produced. Which of the following sentences best describes the connection between the problem presented in the first paragraph and the study described in the second paragraph? (1) The study findings explain how the problem arose; (2) The study examines how the problem impacts the new method; (3) The study shows that the advantage of the new method exceeds the damage caused by the problem; (4) The solution to the problem is incorporated in the study findings.” The SOM present more examples of proximal and distal questions.

Scores on the AAT are composed from a score in the writing assignment and scores in the three multiple-choice sections (verbal reasoning, quantitative reasoning, and English). Each correct answer on the multiple-choice sections is worth one point, there is no penalty for incorrect answers. The raw score for these sections is equal to the number of correct answers. To compute the final AAT score, the administering institute assigns twice as much weight to the verbal reasoning and quantitative reasoning scores as it does to the English score. This policy of the institute reflects considerations of optimizing prediction of academic success. The raw scores of the writing assignment and the score in the multiple-choice sections are then converted to a uniform scale ranging from 200–800.

Study 2

### Participants

One hundred twenty-eight undergraduate students from a large Israeli university (91 women, *M*_age_ = 24.20, *SD* = 2.94) took part in the study and were paid 30 NIS (around US$8) for participation. All participants were native Hebrew speakers. Six participants did not complete the working memory task, and 10 participants did not report their AAT scores. Overall, 112 participants provided both measures. We did not have an estimate of the effect, but planned to be able to detect an effect (main effects of distance and relevance and their interaction) of medium size with a probability of 0.80 at a 5% level of significance. An a-priori power analysis using the G*Power calculator^62^ indicated that a sample size of at least 132 was required. Aiming to meet this goal, data collection continued until the end of the semester. All participants provided informed consent. The study was approved by the institutional review board (IRB) of Tel Aviv University.

### Materials

#### Verbal reasoning

Questions were selected from an online pool of previous academic aptitude tests in Hebrew. We created a proximal and a distal version for each question and added irrelevant information to each version (see Fig. 1 for illustration, or SOM for sample questions). The irrelevant information in the distal and proximal versions was matched in length and linguistic complexity, but questions that included irrelevant information were naturally longer than those that did not. We conducted a pretest to determine the extent to which each question involved psychologically distal/proximal details. In the pretest (*N* = 17, university students, volunteers), 18 questions (nine distal and nine proximal) were presented, and participants rated the extent to which each question was about “a distal (proximal) person, time, or place” on two separate scales (1 = not at all, 7 = extremely). The correlation between the two scales was negative and high (*r* = −0.92). From the 18 pretest questions, we chose 12.

#### Working memory capacity

Visual working memory capacity was measured via the standard change detection task^25,63^. In this task, participants were presented with an array of either four or eight colored squares, that appeared for 150 ms. After a 900 millisecond-long retention interval, one square appeared at one of the previous locations. Participants indicated whether the color of the square is the same as or different from the square presented in the same location in the original array. The task consisted of 20 practice trials, and 120 critical trials. For the detailed description of the task see^58^. Visual-working-memory capacity estimate, *K*_*max*_, was computed by separately averaging accuracy for each array size (four and eight items). These two values were then averaged to form a single parameter with a standard formula^64^, *K*_*max*_ = *S*(*H*-*F*), where *S* is the size of the array, *H* is the observed hit rate (i.e., the proportion of correct answers in trials that presented a change), and *F* is the observed false alarm rate (i.e., the proportion of errors in trials that did not present a change). Higher *K*_*max*_ scores indicate higher capacity (i.e., more items are simultaneously held in memory).

### Procedure

Participants were randomly assigned to either a proximal or a distal condition. Participants were seated individually in a quiet room and were provided with a digital stopwatch and an answer booklet, conditions that resembled a standard AAT. Participants had 12 minutes to complete the verbal reasoning section, and were instructed to answer as accurately as they can as many questions as they can. They were also instructed to guess in case time was over. Each participant was presented with only one version of each question. Relevance was manipulated within participants, such that each participant had six questions with only relevant details, and six questions that included also irrelevant details, presented pseudo-randomly. Participants were informed that the three best performers in the test would receive a 100 NIS cash bonus (around $27). At the end of the experiment, participants provided demographic information and reported their highest attained AAT score. One hundred twenty-three participants (95% of the sample) also completed the change-detection task for another study that took place on the same day, prior to the current study.

Study 3

### Participants

Participants were 1744 examinees, randomly sampled from all the examinees who took the AAT, in two waves. In the first wave, which took place in the summer of 2018, 870 examinees (469 women; *M*_age_ = 22.30, *SD* = 2.95) were sampled. In the second wave, which took place in the summer of 2019, 873 examinees (470 women; *M*_age_ = 21.76 years, *SD* = 2.95) were sampled. While we aimed to reach as many examinees, the organization administering the exam allocated the experimental version on the test according to their operational constraints. Participants signed informed consent as part of the enrollment process to the test.

#### Verbal reasoning

The test was comprised of several chapters that are used for calculating the AAT score and several pilot chapters that do not count toward the final test score but are rather used for operational purposes. Examinees do not know which chapters are the pilot chapters. The experimental questions were incorporated into one of these pilot chapters. We used the same method as in Study 2 with several changes. First, the order of questions in each verbal reasoning section was fixed. In Wave 1 we were able to administer nine new questions, and in Wave 2 we administered 10 of the questions from Study 2. Questions were different in the two waves because the rules of the administering agency do not allow repeating questions across tests.

### Procedure

Participants were randomly assigned to either a proximal or a distal condition. Each examinee received a test form, comprised of several chapters. Each chapter contained 20–23 multiple choice questions with 20 min allotted to complete the chapter. The experimental questions were incorporated into the pilot chapter which did not count toward the examinees’ final AAT score.

### Reporting summary

Further information on research design is available in the Nature Research Reporting Summary linked to this article.

## Supplementary information

Supplementary Material

Reporting Summary

## Data Availability

The raw data and the full analyses code can be found at Open Science Framework repository (https://osf.io/2nvtq).
