# Supplementary material for: Psychological proximity improves reasoning in academic aptitude tests
Source: NPJ Sci Learn. 2023 Apr 29;8:10. doi: 10.1038/s41539-023-00158-x (PMC10148871; doi:10.1038/s41539-023-00158-x)
Supplement: Supplementary file 1 — Supplementary Material [file 41539_2023_158_MOESM1_ESM.pdf]

Supplementary Material

Psychological Proximity Improves Reasoning in Academic Aptitude Tests

Britt Hadar, Maayan Katzir, Sephi Pumpian, Tzur Karelitz, and Nira Liberman

This document includes sample questions from Studies 1, 2, and 3 described in the paper: "Psychological Proximity Improves Reasoning in Academic Aptitude Tests".

We presented participants with verbal reasoning questions and measured accuracy. Each question could be presented either as "distal" or "proximal" and as either "relevant" or irrelevant" regarding its content.

Table of Contents

Sample questions Study 1 1-2

Sample questions Studies 2-3 3-8

Supplementary Material: Psychological Proximity Improves Reasoning.

Sample questions

The original questions were administered in Hebrew. Some of them were translated and are presented here. It is important to note that since psychological distance is dependent on physical location and culture, these questions were adapted to English speaking Americans.

Sample questions Study 1

Far

Beznia's national museum held an exhibition of sculptures by the Dolmanian sculptor Ptolemy. An art critic criticized the museum's management for deciding to hold this exhibition, and wrote, "The museum management's decision can be compared to publishing the poetry of a poet who uses the motif of autumn to portray sadness in a country where the trees do not shed their leaves and there are no migrating birds."

Which of the following statements can be inferred from the critic's words?

(1) Sculptures like Ptolemy's should not be exhibited in a place where the trees do not shed their leaves and there are no migrating birds.
(2) In a place where autumn symbolizes sadness, it makes no sense that sculpture should also symbolize sadness.
(3) In Beznia, the trees shed their leaves and there are migrating birds.
(4) Trees shedding leaves and birds migrating are what make autumn a motif symbolizing sadness in some cultures.

Far

In the year 1952, the following appeared in a literary magazine: "In a 1940 interview, the famous Romanian author M. Petreu announced that he would no longer be writing children's books, but would spend his time writing poetry. While Petreu has indeed stopped writing children's books, he has not yet published a book of poetry".

Assuming that what was written in the magazine is true, which of the following is **impossible**?

(1) All of M. Petreu's books are children's books.
(2) M. Petreu published his first book of poetry in 1953.
(3) All of M. Petreu's books are books of poetry.
(4) M. Petreu published nothing after 1940.

Close

An article written by an American economist states: “The claim that the economic situation of the middle class in the United States has worsened considerably in recent years relative to that of the upper class is the result of focusing on the ever-widening gap in salary between the two classes. However, in order to determine the extent to which the middle class’s situation has worsened, one must consider not only salary scales but also consumption indexes. Results of a survey that examined consumer expenditure in the United States in recent years showed that, in terms of consumption, the gap between the two classes has increased only slightly.”

What is the main claim presented in the above paragraph about the middle class in the United States?

(1) In reality, the salary gap between it and the upper class has increased only slightly in recent years.
(2) In recent years, its economic situation, relative to that of the upper class, has not worsened as much as is generally believed.
(3) When examining its economic situation, more weight should be given to consumption indexes than to salary scales.
(4) The gap between it and the upper class with regard to consumption is greater than the gap between the two with regard to salary.

Close

In recent years a new, mechanized method for separating the seeds of the corn from its pulp was developed. This led to a reduction in the price of corn seeds and their sales rose significantly. As a result, the number of corns grown in the US increased greatly. However, a problem arose: the amount of waste – the corn pulp – that accumulated in the factories separating the seeds grew tremendously. A new study found that adding corn pulp to the fodder for sheep and cows greatly improved their health and the quality and quantity of the milk they produced.

Which of the following sentences best describes the connection between the problem presented in the first paragraph and the study described in the second paragraph?

(1) The study findings explain how the problem arose.
(2) The study examines how the problem impacts the new method.
(3) The study shows that the advantage of the new method exceeds the damage caused by the problem.
(4) The solution to the problem is incorporated in the study findings.

Sample questions Studies 2-3

Question 1:

Far relevant

At a certain time in the evening, there are only four customers sitting in an Indian dhaba restaurant:

Lakshit, Faiyaz, Indrajit and Bhavin.

Given:

- Lakshit is 16 years old.
- Faiyaz drinks only juice.
- Indrajit is 23 years old.
- Bhavin is drinking wine.

Which of the following additional information makes it possible to determine that, at that hour, the law strictly prohibiting alcoholic drinks for people under the age of 18 was not broken in this dhaba restaurant?

(1) Lakshit drinks only tea and Bhavin is 19 years old.

(2) Lakshit drinks only juice and Indrajit drinks beer.

(3) Bhavin is 17 years old and Lakshit drinks only water.

(4) Faiyaz is 15 years old and Indrajit is drinking wine.

Far irrelevant

At a certain time in the evening, there are only four customers sitting in an Indian dhaba restaurant:

Lakshit, Faiyaz, Indrajit and Bhavin, all of whom met long ago in an international online gaming tournament.

Given:

- Lakshit is 16 years old.
- Faiyaz drinks only grape juice.
- Indrajit will turn 24 next year.
- Bhavin is drinking red wine.

Which of the following additional information makes it possible to determine that, at that hour, the law strictly prohibiting alcoholic drinks for people under the age of 18 was not broken in this dhaba restaurant?

Close relevant

At a certain time in the evening, there are only four customers sitting in an American diner.

Jacob, Emma, Ryan and Kaylee.

Given:

- Jacob is 16 years old.
- Emma drinks only juice.
- Ryan is 23 years old.
- Kaylee is drinking wine.

Which of the following additional information makes it possible to determine that, at that hour, the law strictly prohibiting alcoholic drinks for people under the age of 21 was not broken in this diner?

(1) Jacob drinks only Coke and Kaylee is 22 years old.

(2) Jacob drinks only juice and Ryan drinks beer.

(3) Kaylee is 19 years old and Jacob drinks only water.

(4) Emma is 15 years old and Ryan is drinking wine.

Close irrelevant

At a certain time in the evening, there are only four customers sitting in an American diner.

Jacob, Emma, Ryan and Kaylee, all of whom recently met in a local online gaming tournament.

Given:

- Jacob is 16 years old.
- Emma drinks only grape juice.
- Ryan will turn 24 next year.
- Kaylee is drinking red wine.
-

Which of the following additional information makes it possible to determine that, at that hour, the law strictly prohibiting alcoholic drinks for people under the age of 21 was not broken in this diner?

Question 2

Far relevant

Every night, Jacques, a baker, prepares four types of pita bread. Because the different types of dough each require a different amount of time to rise, Jacques first prepares the regular pita, after which he prepares Iraqi pita, then Samaritan pita and finally, Druze pita. Only afterwards does Jacques put all the pita in the oven, doing so in the reverse order from that in which they were prepared.

Which of the following combinations **cannot** exist in the bakery at any given time?

(1) unbaked Iraqi pita on the baker’s table, and Druze pita in the oven.
(2) unbaked Samaritan pita on the baker’s table, and Iraqi pita in the oven.
(3) unbaked regular pita on the baker’s table, and Druze pita in the oven.
(4) unbaked regular pita on the baker’s table, and Samaritan pita in the oven.

Far irrelevant

Every night, Jacques, a baker, prepares four types of pita bread. He needs to make all of them ready for delivery by dawn. The different types of dough each require a different amount of time to rise and Jacques cannot afford to waste time and risk his business. He therefore first prepares the regular pita, after which he prepares Iraqi pita, then Samaritan pita and finally, Druze pita. Only afterwards does Jacques put all the pita in the oven, doing so in the reverse order from that in which they were prepared.

Which of the following combinations **cannot** exist in the bakery at any given time?

Close relevant

Every night, Lucas, a baker, prepares four types of muffins. Because the different types of dough each require a different amount of time to rise, Lucas first prepares the rye muffin, after which he prepares cornbread muffin, then chocolate muffin and finally, butter muffin. Only afterwards does Lucas put all the muffins in the oven, doing so in the reverse order from that in which they were prepared.

Which of the following combinations **cannot** exist in the bakery at any given time?

(1) unbaked Cornbread muffin on the baker’s table, and butter muffin in the oven.
(2) unbaked chocolate muffin on the baker’s table, and Cornbread muffin in the oven.
(3) unbaked rye muffin on the baker’s table, and butter muffin in the oven.
(4) unbaked rye muffin on the baker’s table, and chocolate muffin in the oven.

Close irrelevant

Every night, Lucas, a baker, prepares four types of muffins. He needs to make all of them ready for delivery by dawn. The different types of dough each require a different amount of time to rise and Lucas cannot afford to waste time and risk his business. He therefore first prepares the rye muffin, after which he prepares cornbread muffin, then chocolate muffin and finally, butter muffin. Only afterwards does Lucas put all the muffins in the oven, doing so in the reverse order from that in which they were prepared.

Which of the following combinations **cannot** exist in the bakery at any given time?

Question 3

Far relevant

In ancient Macodi culture, _____ was considered a most important virtue that reflected favorably on the person endowed with it. _____, Macodi mythology attributes _____ to _____.

(1) composure / Yet / the ability to face danger without batting an eyelid / the villain Carvilius, of all people.

(2) kindness / Yet / the willingness to help the needy at any time / the hero Carvilius, of all people.

(3) modesty / Indeed / unparalleled arrogance / the revered hero Carvilius.

(4) physical strength / Indeed / great physical power / the notorious villain Carvilius.

Far irrelevant

In ancient Macodi culture, which prospered in central Asia during the Hellenistic period and has been influenced by a multicultural ethos, _____ was considered a most important virtue that reflected favorably on the person endowed with it. _____, Macodi mythology attributes _____ to _____.

Close relevant

In today American culture, _____ is considered a most important virtue that reflects favorably on the person endowed with it. _____, Stephan King attributes _____ to _____.

(1) composure / Yet / the ability to face danger without batting an eyelid / the villain George Stark, of all people.

(2) kindness / Yet/ the willingness to help the needy at any time / the hero George Stark, of all people.

(3) modesty / Indeed / unparalleled arrogance / the revered hero George Stark.

(4) physical strength / Indeed / great physical power / the notorious villain George Stark.

Close irrelevant

In modern American culture, which is prominent in United States and north America and is influenced by a multicultural ethos, ____ is considered a most important virtue that reflects favorably on the person endowed with it. _____, Stephan King attributes _____ to _____.

(1) composure / Yet / the ability to face danger without batting an eyelid / the villain George Stark, of all people.

(2) kindness / Yet/ the willingness to help the needy at any time / the hero George Stark, of all people.

(3) modesty / Indeed / unparalleled arrogance / the revered hero George Stark.

(4) physical strength / Indeed / great physical power / the notorious villain George Stark.

Question 4

Far relevant

In his new study, Abbas claims that the declarations made by Roman statesmen were generally _____ succeeded _____ the citizens of ancient Rome _____ modern historians, who regard those assertions as _____.

(1) Empty slogans that / in deceiving not only / but also / a well-structured and reasoned political doctrine

(2) meaningful statements that were given careful consideration and they / in making a deep impression not only on / but also on / worthless

(3) hollow words that / in deceiving / but not / constituting a deep political philosophy

(4) meaningless, but they / despite their meaninglessness, in making inroads with / as well as with / empty and useless

---

Far irrelevant

In his new controversial study, Prof. Abbas El Gamal from Cairo University claims that the declarations made by Roman statesmen were generally _____ succeeded _____ the citizens of ancient Rome _____ modern historians and political scientists, who regard those assertions as _____.

(1) Empty slogans that / in deceiving not only / but also / a well-structured and reasoned political doctrine

(2) meaningful statements that were given careful consideration and they / in making a deep impression not only on / but also on / worthless

(3) hollow words that / in deceiving / but not / constituting a deep political philosophy

(4) meaningless, but they / despite their meaninglessness, in making inroads with / as well as with / empty and useless

---

Close relevant

In his new study, Andrew claims that the declarations made by the White House Press Secretary were generally _____ succeeded _____ the citizens of the United States _____ modern historians, who regard those assertions as _____.

(1) Empty slogans that / in deceiving not only / but also / a well-structured and reasoned political doctrine
(2) meaningful statements that were given careful consideration and they / in making a deep impression not only on / but also on / worthless
(3) hollow words that / in deceiving / but not / constituting a deep political philosophy
(4) meaningless, but they / despite their meaninglessness, in making inroads with / as well as with / empty and useless

---

Close irrelevant

In his new controversial study, Prof. Andrew S. Ross from University of Minnesota claims that the declarations made by the White House Press Secretary were generally _____ succeeded _____ the citizens of the United States _____ modern historians and political scientists, who regard those assertions as _____.

(1) Empty slogans that / in deceiving not only / but also / a well-structured and reasoned political doctrine
(2) meaningful statements that were given careful consideration and they / in making a deep impression not only on / but also on / worthless
(3) hollow words that / in deceiving / but not / constituting a deep political philosophy
(4) meaningless, but they / despite their meaninglessness, in making inroads with / as well as with / empty and useless

---
