# Supplementary material for: Psychological proximity improves reasoning in academic aptitude tests
Source: NPJ Sci Learn. 2023 Apr 29;8:10. doi: 10.1038/s41539-023-00158-x (PMC10148871; doi:10.1038/s41539-023-00158-x)
Supplement: Supplementary file 2 — Reporting Summary [file 41539_2023_158_MOESM2_ESM.pdf]

nature portfolio

Corresponding author(s): Britt Hadar NPJSCILEARN-00633R1

Last updated by author(s): 02/14/2023

# Reporting Summary

Nature Portfolio wishes to improve the reproducibility of the work that we publish. This form provides structure for consistency and transparency in reporting. For further information on Nature Portfolio policies, see our Editorial Policies and the Editorial Policy Checklist.

## Statistics

For all statistical analyses, confirm that the following items are present in the figure legend, table legend, main text, or Methods section.

| n/a | Confirmed | |
|---|---|---|
| ☐ | ☒ | The exact sample size (*n*) for each experimental group/condition, given as a discrete number and unit of measurement |
| ☐ | ☒ | A statement on whether measurements were taken from distinct samples or whether the same sample was measured repeatedly |
| ☐ | ☒ | The statistical test(s) used AND whether they are one- or two-sided<br>*Only common tests should be described solely by name; describe more complex techniques in the Methods section.* |
| ☐ | ☒ | A description of all covariates tested |
| ☐ | ☒ | A description of any assumptions or corrections, such as tests of normality and adjustment for multiple comparisons |
| ☐ | ☒ | A full description of the statistical parameters including central tendency (e.g. means) or other basic estimates (e.g. regression coefficient) AND variation (e.g. standard deviation) or associated estimates of uncertainty (e.g. confidence intervals) |
| ☐ | ☒ | For null hypothesis testing, the test statistic (e.g. *F*, *t*, *r*) with confidence intervals, effect sizes, degrees of freedom and *P* value noted<br>*Give P values as exact values whenever suitable.* |
| ☒ | ☐ | For Bayesian analysis, information on the choice of priors and Markov chain Monte Carlo settings |
| ☐ | ☒ | For hierarchical and complex designs, identification of the appropriate level for tests and full reporting of outcomes |
| ☐ | ☒ | Estimates of effect sizes (e.g. Cohen's *d*, Pearson's *r*), indicating how they were calculated |

*Our web collection on statistics for biologists contains articles on many of the points above.*

## Software and code

Policy information about availability of computer code

| | |
|---|---|
| Data collection | Data collection was carried on pen and paper tests both in lab and in the field. |
| Data analysis | All data and analysis code are available at Open Science Repository (osf.io/2nvtq/?view_only=30d4747b54a243ea90436f7661891365). Data were analyzed using R, version 4.1.2 (R Core Team, 2021) and the package ggplot, version 3.3.5. In all studies we ran a generalized linear mixed model (GLMM) using the glmer function (family = binomial) in the lme4 package in R version 1.1-30 [47]. |

For manuscripts utilizing custom algorithms or software that are central to the research but not yet described in published literature, software must be made available to editors and reviewers. We strongly encourage code deposition in a community repository (e.g. GitHub). See the Nature Portfolio guidelines for submitting code & software for further information.

## Data

Policy information about availability of data

All manuscripts must include a data availability statement. This statement should provide the following information, where applicable:

- Accession codes, unique identifiers, or web links for publicly available datasets
- A description of any restrictions on data availability
- For clinical datasets or third party data, please ensure that the statement adheres to our policy

The raw data and the full analyses code can be found at Open Science Framework repository (https://osf.io/2nvtq/?view_only=30d4747b54a243ea90436f7661891365)

# Human research participants

Policy information about studies involving human research participants and Sex and Gender in Research.

| | |
|---|---|
| Reporting on sex and gender | Gender was self reported.<br>Study 1: Each sample had a different percentage of women as indicated at the SOM.<br>Study 2: One hundred twenty-eight undergraduate students from a large Israeli university (91 women, Mage = 24.20, SD = 2.94) took part in the study.<br>Study 3: Participants were 1,744 examinees, randomly sampled from all the examinees who took the AAT in two waves. In the first wave, which took place in the summer of 2018, 870 examinees (469 women; Mage = 22.30, SD = 2.95) were sampled. In the second wave, which took place in the summer of 2019, 873 examinees (470 women; Mage = 21.76 years, SD = 2.95) were sampled. |
| Population characteristics | Study 1 and 3. Examinees in an academic aptitude test in Israel.<br>Study 2: Students in Tel Aviv University. |
| Recruitment | Study 1: Random sample<br>Study 2: Recruitment through ads at the university<br>Study 3: representative sample of all examinees who took part in the exam. |
| Ethics oversight | Tel Aviv University Institutional review board and National Institute for Testing and Eveluation (NITE). |

Note that full information on the approval of the study protocol must also be provided in the manuscript.

# Field-specific reporting

Please select the one below that is the best fit for your research. If you are not sure, read the appropriate sections before making your selection.

☐ Life sciences ☒ Behavioural & social sciences ☐ Ecological, evolutionary & environmental sciences

For a reference copy of the document with all sections, see nature.com/documents/nr-reporting-summary-flat.pdf

# Behavioural & social sciences study design

All studies must disclose on these points even when the disclosure is negative.

| | |
|---|---|
| Study description | Data are quantitative |
| Research sample | Study 1: Examinees taking an aptitude test in Israel. Each sample had a different percentage of women as indicated at the SOM.<br>Study 2: Students at Tel Aviv University. One hundred twenty-eight undergraduate students from a large Israeli university (91 women, Mage = 24.20, SD = 2.94) took part in the study.<br>Study 3: Examinees taking an aptitude test in Israel. Participants were 1,744 examinees, randomly sampled from all the examinees who took the AAT in two waves. In the first wave, which took place in the summer of 2018, 870 examinees (469 women; Mage = 22.30, SD = 2.95) were sampled. In the second wave, which took place in the summer of 2019, 873 examinees (470 women; Mage = 21.76 years, SD = 2.95) were sampled.<br><br>We examined different factors that might affect performance in aptitude tests, therefore examinees and students are the most relevant population for this purpose. |
| Sampling strategy | Study 1. Random<br>Study 2. Convenience<br>Study 3. Representative |
| Data collection | In all studies experimenters were blind to the study conditions. In all studies responses were recorded by pen and paper. |
| Timing | Study 1. 2014-2017. We collected relevant test items that manipulated psychological distance post hoc.<br>Study 2. Data was collected in the lab during spring semester of 2018.<br>Study 3. Data was collected in two waves of aptitude tests in the field - in the summer of 2018 and of 2019. |
| Data exclusions | Study 1 and 3. No exclusions<br>Study 2. Out of 128 participants, six participants did not complete the working memory task, and 10 participants did not report their AAT scores. Overall, 112 participants provided both measures. |
| Non-participation | No drop-outs. |

| | |
|---|---|
| Randomization | Allocation to groups was random. |

# Reporting for specific materials, systems and methods

We require information from authors about some types of materials, experimental systems and methods used in many studies. Here, indicate whether each material, system or method listed is relevant to your study. If you are not sure if a list item applies to your research, read the appropriate section before selecting a response.

## Materials & experimental systems

| n/a | Involved in the study |
|---|---|
| ☒ | ☐ Antibodies |
| ☒ | ☐ Eukaryotic cell lines |
| ☒ | ☐ Palaeontology and archaeology |
| ☒ | ☐ Animals and other organisms |
| ☒ | ☐ Clinical data |
| ☒ | ☐ Dual use research of concern |

## Methods

| n/a | Involved in the study |
|---|---|
| ☒ | ☐ ChIP-seq |
| ☒ | ☐ Flow cytometry |
| ☒ | ☐ MRI-based neuroimaging |
